# Effects of grape pomace on growth performance, serum biochemical indices, amino acid composition, and rumen microbial diversity in Dorper × Hu hybrid sheep

**DOI:** 10.3389/fvets.2025.1717637

**Published:** 2026-01-20

**Authors:** Xinchang Sun, Xia Zhang, Meimei Teng, Yuanqiu Li, Jiangjiao Qi, Tursunay Mamtimin, Wenda Wu, Jiangchun Wan

**Affiliations:** 1 Xinjiang Key Laboratory of Grassland Resources and Ecology, College of Grassland Science, Xinjiang Agricultural University, Urumgi, China; 2 Joint Research Center for Foodborne Functional Factors and Green Preparation, School of Food and Biological Engineering, Engineering Research Center of Bio-process, Ministry of Education, Hefei University of Technology, Hefei, China; 3 Department of Chemistry, Faculty of Science, University of Hradec Kralove, Hradec Kralove, Czechia

**Keywords:** Dorper × Hu hybrid sheep, grape pomace, growth performance, rumen microorganism, serum biochemical indices

## Abstract

**Introduction:**

Grape pomace (GP), a byproduct of wine-making, is rich in nutrients and bioactive compounds. However, its effects on the health and rumen microbial diversity of fattening rams remain insufficiently studied. This study investigated the impact of dietary GP supplementation on Dorper × Hu hybrid sheep.

**Methods:**

Eighteen male F1 Dorper× Hu hybrid sheep were randomly divided into three groups. They were fed complete pelleted diets containing 0% (control), 15%, or 20% GP for 60 days. Growth performance, serum biochemical indices, amino acid profiles in the longissimus dorsi muscle, and rumen bacterial composition were evaluated.

**Results:**

The key findings were: (1) GP inclusion did not negatively affect growth performance. (2) It significantly increased serum total protein levels and total antioxidant capacity, and elevated the content of essential amino acids in the muscle. (3) While overall rumen microbial diversity was not altered, GP supplementation specifically reduced the abundance of certain potentially harmful bacteria, such as Treponema.

**Discussion:**

The results demonstrate that incorporating GP into the diet can enhance antioxidant status and improve muscle amino acid profiles in Dorper × Hu hybrid sheep, without compromising production performance. The selective reduction in specific rumen bacteria suggests a modulatory effect. This study provides a theoretical foundation for the effective utilization of GP in complete pellet feeds for ruminants.

## 1 Introduction

Grape, a fruit of deciduous lianas in the Vitaceae family, has the largest cultivation area worldwide. In recent years, the continued expansion of vineyard acreage has led to a corresponding annual increase in grape production. Grape pomace (GP), a key by-product of wine-making, is composed of skins, stems, and seeds (1). It is rich in nutrients and bioactive compounds, such as anthocyanins, resveratrol, and flavonoids, which are associated with multiple health benefits (2). Scientific and reasonable development of GP could mitigate feed costs and enhance the utilization of unconventional feed resources to improve forage shortages.

While previous GP research has centered on monogastric animals and large ruminants, studies on fattening rams remain limited (3, 4). Key indicators such as production performance, blood parameters, and meat amino acid content are vital for assessing sheep farming profitability. Evidence suggests that GP, particularly when treated with N. sitophila, is a beneficial supplementary feed for ruminants (5). For instance, Guelfi et al. reported that a 5% GP diet was safe for sheep, modified milk composition via nitrogen metabolism, and did not affect meat quality (6). Another study found that 2% dried GP improved crude protein digestibility in sheep (7). Given that the rumen microbiota plays a pivotal role in ruminant digestion and metabolism (8), and considering that GP is known to influence this microbial diversity (9, 10), its role in sheep nutrition warrants further investigation.

There is scarcity of research on the impact of GP on the health and rumen microbial diversity of Dorper × Hu Hybrid sheep. Therefore, this study was conducted to determine the effects of GP on production performance, serum biochemical indices, amino acid profiles in the longissimus dorsi muscle, and rumen bacterial diversity in Dorper × Hu Hybrid sheep.

## 2 Materials and methods

### 2.1 Experimental materials

GP used in the experiment, sourced from Cabernet Sauvignon wine grapes provided by Xinjiang CITIC Guoan Wine Industry Co., Ltd., was an unprocessed raw material containing skins, pulp, and seeds. The nutritional components were determined using standard methods: crude protein (CP) content was determined by the Kjeldahl method and measured 13.10%; ether extract (EE) content was determined by Soxhlet extraction and measured 13.30%; neutral detergent fiber (NDF) and acid detergent fiber (ADF) contents were determined by the Van Soest method and measured 44.74 and 39.42%, respectively; crude ash content was determined by ashing and measured 4.60%.

### 2.2 Experimental animals and feeding management

A total of 18 3-month-old healthy Dorper × Hu hybrid male lambs with similar body weight [(28.64 ± 0.32) kg] were selected for this study, which was conducted at a farmer’s residence in Xiatougong Village, Daxiqu Town, Changji City, Xinjiang, from April 7th to June 15th, 2024. The experiment consisted of an 8-day pre-feeding period followed by a 60-day formal feeding trial. Using a completely randomized design, the sheep were randomly assigned to three dietary treatments with six replicates (pens) per treatment and one lamb per pen. The experimental diets were formulated to be isoenergetic and isonitrogenous, containing 0% (CK), 15%, or 20% GP, as detailed in Table 1.

**Table 1:** Basal diet composition and nutrient level.

Items	Group		
	CK	15% GP	20% GP
Ingredients			
Corn	18.00	17.55	16.60
Wheat bran	9.50	9.00	9.00
Sunflower seed meal	7.65	7.00	6.25
Capsicum meal	2.00	2.00	1.80
Cottonseed meal	1.00	1.00	1.00
Squash seed	13.00	13.10	13.10
Premix^1^	1.00	1.00	1.00
Soybean meal	11.00	11.55	11.30
NaCl	1.00	1.00	1.00
Corn husk	1.00	3.00	3.00
Alfalfa hay	8.10	5.80	6.85
Maize straw	24.00	9.00	4.00
Grape pomace		15.00	20.00
Wheat straw	1.75	2.00	2.00
Cotton straw	1.00	2.00	3.10
Total	100.00	100.00	100.00
Nutrient levels			
Dry matter	91.16	89.64	89.72
Crude protein	14.12	14.16	14.14
ME^2^, MJ/kg	8.51	8.60	8.62

1 Premix: 0.1 g Cu, 1 g Fe, 1.5 g Zn, 0.5 g Mn, 5.0 mg Se, 12.0 mg I, 5 mg Co, 200 mg Ca, 15 mg P, 120000 IU vitamin A, 10000 IU vitamin D, 750 IU vitamin E.

2 ME was a calculated value according to NY/T816—2021, while the others were measured values.

Before the experiment, all sheep pens were thoroughly cleaned and sanitized. The sheep were dewormed, vaccinated, and ear-tagged for identification. They were housed in individual pens according to their treatment groups and fed twice daily at 09:00 and 19:00 with free access to water. Pens were cleaned daily, and the health status of the sheep was regularly monitored.

### 2.3 Materials production performance determination

At the beginning and end of the formal trial, all sheep were fasted for 12 h before their initial and final body weights were recorded. Throughout the experimental phase, daily feed intake was recorded fby group to calculate the average daily feed intake (ADFI), average daily gain (ADG), and the feed-to-gain ratio (F/G). Upon completion of the trial, was randomly selected from each replicate and slaughtered to determine the carcass yield.

After the end of the experimental period, three sheep per group were randomly selected after a 24-h fast followed by 1 h of water deprivation. From each animal, 30 mL of blood was collected from the jugular venipuncture into vacuum tubes. The blood samples were centrifuged at 4,000 r/min for 10 min at 4 °C to separate the serum. The resulting serum was then transferred into EP tubes and stored at −20 °C pending analysis.

Serum lipid metabolites, including urea nitrogen, glucose, total protein, albumin, globulin, triglyceride, total cholesterol, high-density lipoprotein cholesterol, low-density lipoprotein cholesterol, and lactate dehydrogenase, were quantified using a fully automated biochemical analyzer (Beckman AU5821, United States) (11). The activities of glutathione peroxidase (GSH-Px), catalase (CAT), total superoxide dismutase (T-SOD), total antioxidant capacity (T-AOC), and the concentration of malondialdehyde (MDA) were assessed using commercial assay kits (Elabscience Biotechnology Co., Ltd., Wuhan, China). Specifically, GSH-Px, MDA, and CAT were determined by colorimetry, while T-SOD was measured using the WST-1 method.

### 2.4 Determination of rumen bacterial community

Fresh rumen fluid was collected immediately post-slaughter and the microbial delivered to Shanghai Personalbio Biotechnology Co., Ltd. for amplicon sequencing and microbial genomic DNA was extracted using a commercial kit (MP Biomedicals, Cat. No. 116564384). The V3-V4 hypervariable regions of the bacterial 16S rRNA gene were amplified with universal prokaryotic primers: 338F (5’-ACTCCTACGGGAGGCAGCA-3′) and 806R (5’-GGACTACHVGGGTWTCTAAT-3′). Sequencing adapters were ligated to the primer ends. The PCR amplification was performed in a 25 μL reaction mixture containing 0.25 μL of Q5 high-fidelity DNA polymerase, 5 μL of 5 × Reaction Buffer, 5 μL of 5 × High GC Buffer, 2 μL of dNTPs (10 mM), 2 μL of template DNA, 1 μL of each forward and reverse primer (10 μM), and 8.75 μL of ddH₂O. The thermal cycling conditions were as follows: initial denaturation at 98 °C for 5 min; 25 cycles of denaturation at 98 °C for 30 s, annealing at 52 °C for 30 s, and extension at 72 °C for 45 s; with a final extension at 72 °C for 5 min. The PCR products were verified by 2% agarose gel electrophoresis. The target bands were excised and purified using the Axygen Gel Extraction Kit. The purified amplicons were quantified and normalized to construct the sequencing libraries. After passing quality control, the libraries were subjected to high-throughput sequencing on an Illumina NovaSeq platform (PE 250) to generate raw paired-end reads for subsequent bioinformatic analysis.

Alpha diversity indices (Chao1, Shannon, and Simpson) for the rumen microbiota were calculated using the QIIME toolkit.

### 2.5 Statistical analysis of data

After organizing the data in Excel, statistical analysis was conducted using SPSS 27.0, following verification of assumptions for normality and homogeneity of variances. Results were presented as mean ± standard error. One-way ANOVA was performed to evaluate the effects of different levels of GP substitution on the production performance, serum biochemical indicators,organic acid composition in the musculature of the longissimus dorsi of Dorper × Hu sheep. LSD (Least Significant Difference) was used for inter-group comparisons. Differences were considered significant at *p* < 0.05, and highly significant at *p* < 0.01.

## 3 Results

### 3.1 Effects of GP on the production performance of Dorper × Hu sheep

As indicated in Table 2, the addition of GP in the diets of Dorper × Hu sheep did not significantly affect their ADG, ADFI, or F/G. While the 20% GP treatment increased ADG by 14.29% and reduced the feed-to-weight ratio by 18.51%, these changes were not significant. Similarly, the 15% GP treatment resulted in a reduction in the feed-to-weight ratio by 9.35%, but this difference was also not significant.

**Table 2:** Effects of GP on the growth performance of Dorper × Hu sheep.

Items	Grape residue levels			*p*-value
	CK	15% GP	20% GP	
Initial weight, kg	28.22 ± 0.21	28.98 ± 0.91	27.88 ± 0.16	0.41
Final weight, kg	37.22 ± 0.37	37.71 ± 0.65	38.29 ± 0.56	0.43
Average daily gain, kg/d	0.17 ± 0.01	0.18 ± 0.02	0.20 ± 0.02	0.46
Average daily feed intake, kg/d	1.42 ± 0.05	1.49 ± 0.04	1.42 ± 0.08	0.62
F/G	9.41 ± 0.50	8.53 ± 0.73	8.77 ± 0.74	0.65
Carcass weight, kg	17.52 ± 1.02	17.95 ± 1.32	18.32 ± 1.67	0.92
Dressing percentage, %	43.42 ± 0.50	41.93 ± 1.97	45.32 ± 1.15	0.28

*Results are expressed as “mean ± standard error.” *p* > 0.05 was not significant.

### 3.2 Effects of GP levels on serum biochemical indicators of Dorper × Hu sheep

As indicated in Table 3, compared to the CK group, the supplementation with GP significantly increased the serum levels of total protein, albumin, and globulin in both the 15 and 20% GP treatments. Similarly, total cholesterol was significantly elevated in both GP treatment groups. However, no significant differences were observed in HDL-C, LDL-C, glucose, triglycerides, lactate dehydrogenase, or total superoxide dismutase levels among the groups.

**Table 3:** Effects of GP Levels on serum biochemical indicators of Dorper × Hu sheep.

Items	Grape residue levels			*p*-value
	CK	15%GP	20%GP	
Initial weight, kg	28.22 ± 0.21	28.98 ± 0.91	27.88 ± 0.16	0.41
Urea nitrogen, mmol/L	0.71 ± 0.03^b^	0.85 ± 0.11^b^	1.45 ± 0.18^a^	0.01
Glucose, mmol/L	6.16 ± 0.23	5.59 ± 0.53	6.17 ± 1.08	0.81
Total protein, g/L	50.27 ± 0.55^C^	72.40 ± 1.06^B^	77.43 ± 1.26^A^	<0.01
Albumin, g/L	19.63 ± 0.15^B^	27.33 ± 0.23^A^	27.37 ± 1.18^A^	<0.01
Globulins, g/L	30.30 ± 0.14^C^	45.07 ± 1.00^B^	50.07 ± 0.81^A^	<0.01
Albumin/Globulins	0.64 ± 0.01^a^	0.61 ± 0.01^ab^	0.55 ± 0.03^b^	0.03
Triglycerides, mmol/L	0.35 ± 0.04	0.41 ± 0.01	0.27 ± 0.07	0.18
Total cholesterol, mmol/L	0.76 ± 0.01^B^	1.17 ± 0.00^A^	1.14 ± 0.01^A^	<0.01
high density lipoprotein cholesterol, mmol/L	0.52 ± 0.02	0.89 ± 0.10	0.67 ± 0.13	0.09
low density lipoprotein cholesterol, mmol/L	0.27 ± 0.01	0.45 ± 0.05	0.30 ± 0.07	0.08
Lactate dehydrogenase, U/L	366.00 ± 12.17	465.67 ± 61.13	401.67 ± 58.33	0.41
Total superoxide dismutase, U/mL	52.14 ± 4.84	59.08 ± 4.40	58.38 ± 3.91	0.50
Catalase levels, U/mL	170.87 ± 1.87^B^	212.40 ± 4.89^A^	176.83 ± 5.52^B^	<0.01
Total antioxidant capacity, U/mL	1.53 ± 0.15^B^	1.84 ± 0.06^B^	2.83 ± 0.49^A^	<0.01
Malondialdehyde, μmol/L	7.75 ± 0.20^A^	2.27 ± 0.26^B^	2.69 ± 0.25^B^	<0.01
Glutathione peroxidase, U	941.67 ± 7.68^C^	1036.90 ± 7.68^B^	1468.45 ± 13.20^A^	<0.01

*Results are expressed as mean ± standard error. Different peer letters were significantly different, with lowercase letters indicating *p* < 0.05 and upper case letters indicating *p* < 0.01.

Regarding antioxidant capacity, CAT was significantly higher only in the 15% GP group compared to the CK and 20% GP groups. T-AOC was significantly increased in the 20% GP group. In contrast, MDA levels were significantly decreased in both GP treatments. GSH-Px activity was significantly enhanced in both GP groups, with the 20% GP group showing the greatest improvement.

### 3.3 Effects of GP substitution level on amino acid composition in the longissimus thoracis of Dorper × Hu sheep

As shown in Table 4, a total of 16 amino acids were detected in all experimental groups, comprising 7 essential amino acids (EAAs) and 9 non-essential amino acids (NEAAs). The total EAA content in the sheep fed with 20% GP was significantly higher than in the CK group and the 15% GP group. The total NEAA content in the GP-added treatments showed an increasing trend, but the difference was not statistically significant.

**Table 4:** Effect of GP on amino acid content in the longissimus thoracis of Dorper × Hu Sheep.

Items	Grape residue levels			*p*-value
	CK	15% GP	20% GP	
Methionine	5.57 ± 0.11	4.94 ± 0.24	5.46 ± 0.27	0.12
Valine	24.88 ± 0.80^B^	28.71 ± 0.88^A^	29.93 ± 0.61^A^	<0.01
Lysine	10.80 ± 0.76	10.11 ± 0.17	11.86 ± 0.30	0.05
Isoleucine	4.75 ± 0.36	5.94 ± 0.42	5.08 ± 0.34	0.09
Phenylalanine	11.62 ± 0.59	11.73 ± 0.58	13.30 ± 0.47	0.07
Leucine	9.03 ± 0.42^b^	9.70 ± 0.26^ab^	11.06 ± 0.70^a^	0.02
Threonine	11.37 ± 0.82	11.50 ± 0.85	13.29 ± 0.84	0.21
Total essential amino acids	78.01 ± 2.14^B^	82.62 ± 2.75^B^	89.98 ± 2.41^A^	<0.01
Histidine	150.58 ± 0.83	160.34 ± 0.64	167.45 ± 0.23	0.17
Aspartic acid#	6.74 ± 1.14	10.56 ± 1.97	6.90 ± 1.08	0.13
Glycine#	0.36 ± 0.03^B^	0.25 ± 0.03^C^	0.49 ± 0.04^A^	<0.01
Glutamic acid#	21.77 ± 3.30^ab^	27.35 ± 2.28^a^	16.34 ± 0.99^b^	0.01
Proline	50.5 ± 4.17^a^	39.32 ± 1.37^b^	44.34 ± 2.22^ab^	0.04
Alanine	9.73 ± 0.55^b^	10.63 ± 0.35^ab^	12.43 ± 0.92^a^	0.02
Serine	27.83 ± 1.22^B^	23.38 ± 0.99^C^	33.34 ± 1.72^A^	<0.01
Arginine	34.51 ± 3.30	30.44 ± 1.65	32.39 ± 1.13	0.45
Tyrosine	23.88 ± 0.71^B^	27.93 ± 1.36^A^	28.83 ± 0.36^A^	<0.01
Total non-essential amino acids	325.89 ± 16.91	330.20 ± 6.88	342.60 ± 4.07	0.53

Regarding flavor amino acids, the glycine content in the 20% GP treatment was significantly higher than in the CK treatment. The glutamic acid content in the 15% GP treatment was significantly higher than that in both the CK and the 20% GP groups.

### 3.4 Effects of GP on rumen bacterial flora composition of Dorper × Hu sheep

**Figure 1:**
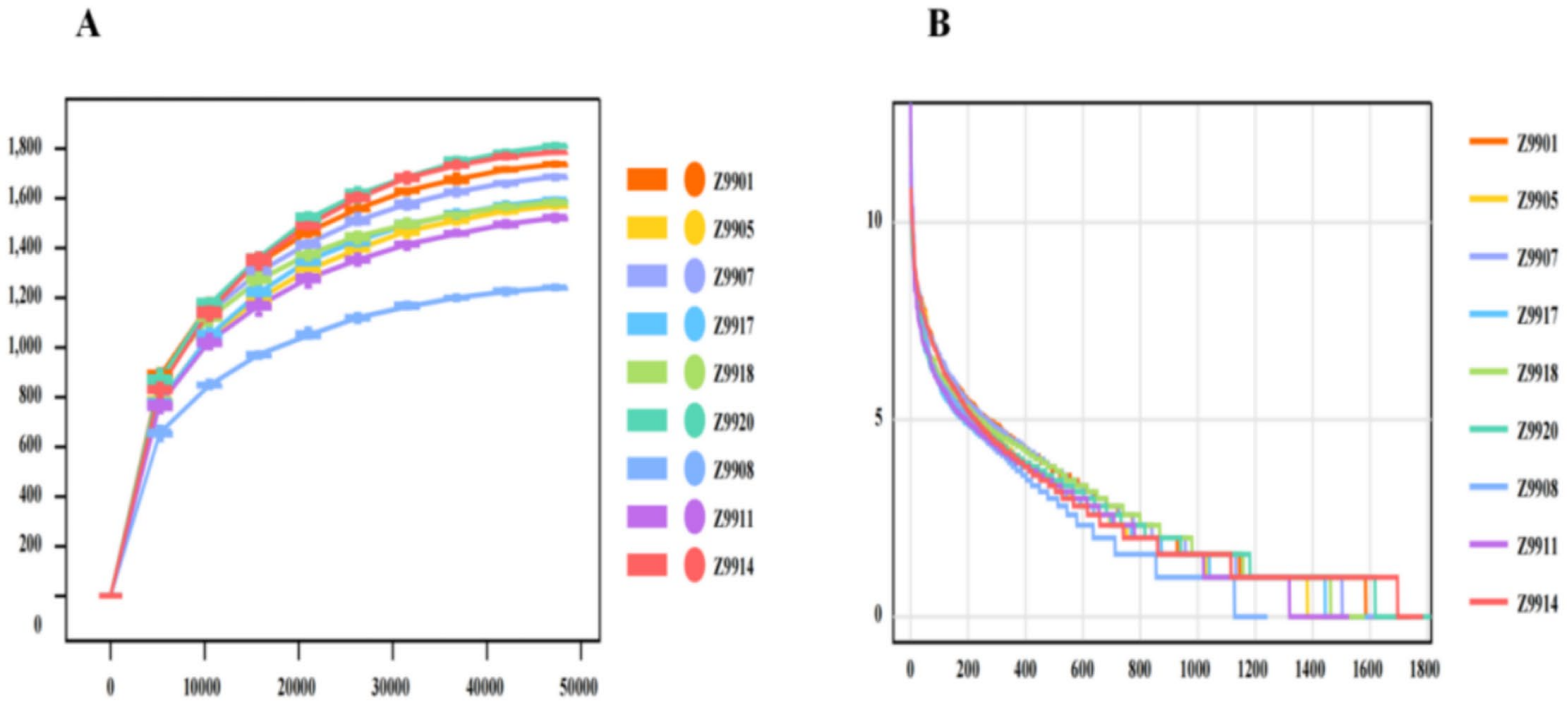
Composition of the rumen microflora in fattening rams. **(A)** Rarefaction curve. **(B)** Abundance rank curve. Z9901, Z9905, and Z9907 were employed as CK replicates; Z9917, Z9918, and Z9920 constituted the 15% GP group; and Z9908, Z9911, and Z9914 represented the 20% GP group.

The plateau of rarefaction curves (Figure 1A) indicates that the sequencing depth was sufficient to capture the majority of microbial diversity. The abundance rank curve (Figure 1B) reflects the evenness of community composition, where flatter lines indicate greater evenness.

**Figure 2:**
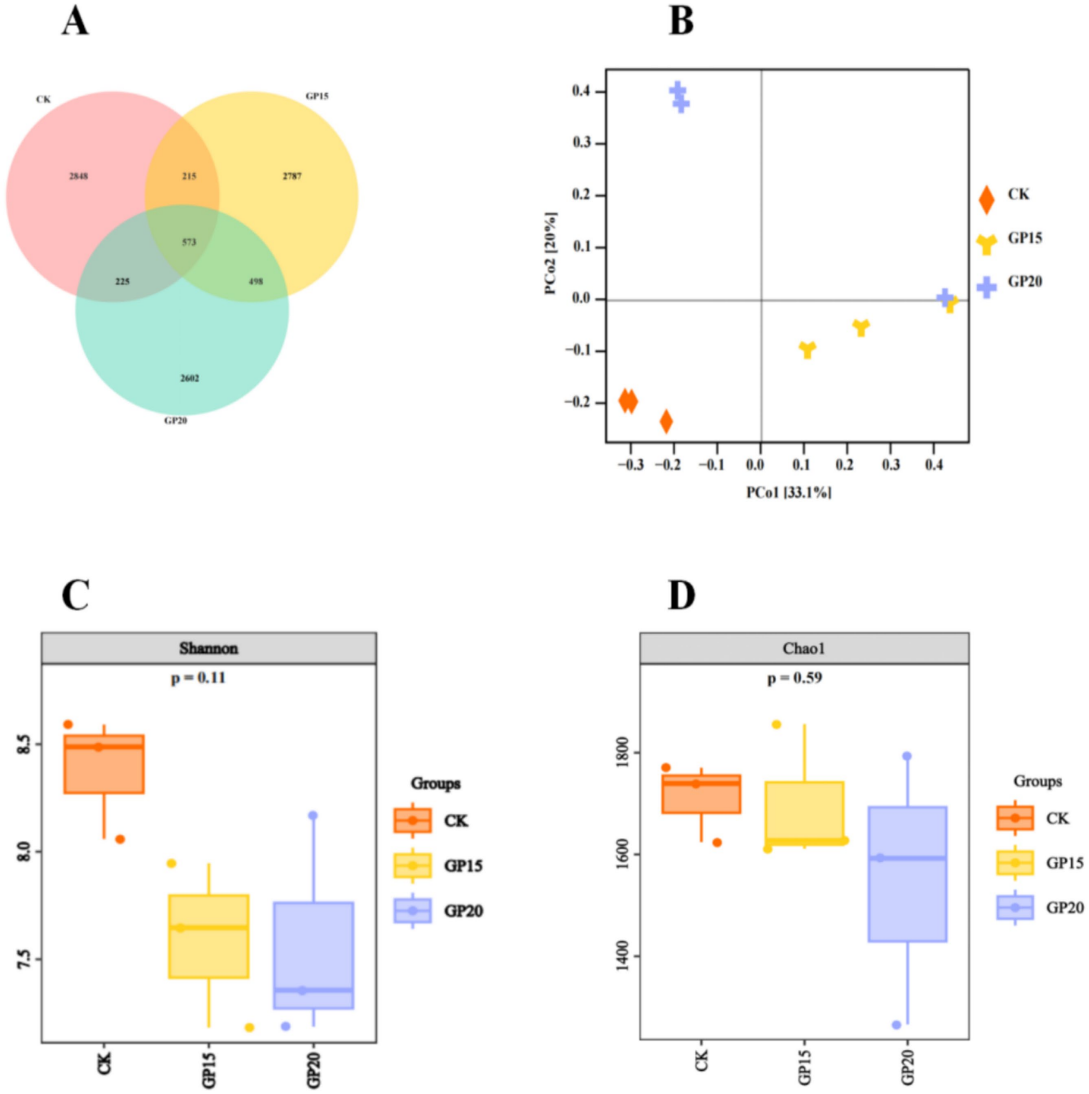
Rumen microflora composition in fattening rams. **(A)** Venn plots of OTU distribution among groups. **(B)** Principal coordinate analysis (PCoA) based on weighted UniFrac distances, showing distinct clustering of bacterial communities. **(C)** Shannon and **(D)** Chao1 alpha-diversity indices. Data in **(C,D)** were analyzed by univariate ANOVA; no significant differences were found (*p* > 0.05).

A total of 8,237 OTUs were identified across the three groups (Figure 2A). The CK, 15% GP, and 20% GP groups contained 2,848, 2,787, and 2,602 OTUs, respectively, with only 573 OTUs (6.96%) shared among all groups. Principal coordinate analysis (PCoA) based on weighted UniFrac distances showed that each group formed distinct clusters (Figure 2B), suggesting significant differences in rumen bacterial community composition due to GP inclusion.

To assess the impact of GP levels on the alpha diversity of rumen bacteria, indicators such as Chao1, Observed_species, Faith_pd, Shannon, and Simpson were measured. As shown in Figures 2C,D, the inclusion of GP did not significantly affect the Chao1 and Shannon indices (*p* > 0.05), and no consistent trend was observed. Similarly, other alpha diversity indices including Observed_species, Faith_pd, and Simpson were also not significantly affected by GP inclusion.

### 3.5 Effects of GP on the abundance of rumen flora in Dorper × Hu sheep

**Figure 3:**
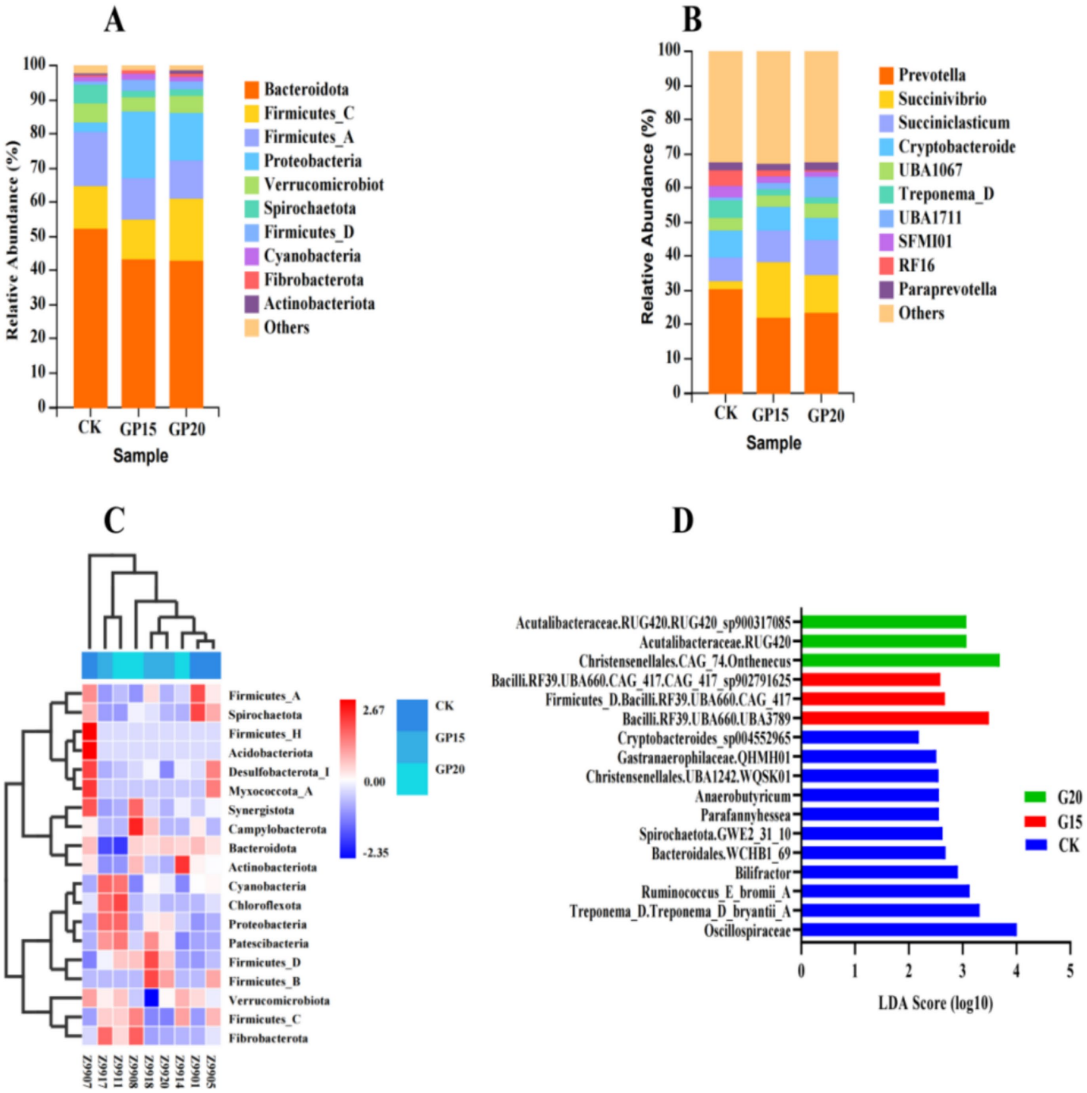
Structural changes in the rumen bacterial community of fatten in grams in response to dietary grape pomace supplementation. **(A)** Relative abundance of bacterial communities at the phylum level. **(B)** Relative abundance of the dominant bacterial genera. **(C)** Heatmap of bacterial phyla composition across different samples. **(D)** LEfSe analysis identifying differentially abundant bacterial taxa among groups (LDA score>2.0).

Based on the analysis of rumen microbial composition, dietary supplementation with GP induced substantial structural shifts in the bacterial community of fattening rams. At the phylum level, the microbiota was predominantly composed of Bacteroidetes and Firmicutes across all groups (Figure 3A). Notably, GP inclusion led to an increase in the relative abundance of Firmicutes and a corresponding decrease in Bacteroidetes, resulting in an elevated Firmicutes-to-Bacteroidetes ratio in the 15% GP and 20% GP groups compared to the control.

This structural reorganization was further corroborated by the genus-level composition (Figure 3B), where Prevotella remained the most abundant genus, though its relative abundance varied among treatments. The heatmap at the phylum level (Figure 3C) reinforced these findings, showing distinct clustering of samples according to dietary treatment and visually confirming the shift in major bacterial phyla.

LEfSe analysis (Figure 3D) identified statistically significant biomarkers enriched in each group, demonstrating that GP supplementation significantly altered the rumen microbiota by promoting distinct bacterial taxa in a dose-dependent manner. Specifically, the control group exhibited the highest number of unique biomarkers, while the 15% GP and 20% GP groups each developed their own characteristic microbial signatures.

In conclusion, GP supplementation did not merely modulate the abundance of individual taxa but systematically reshaped the overall rumen bacterial ecosystem, favoring a community structure potentially associated with enhanced energy harvest efficiency.

## 4 Discussion

In livestock production, the primary objective is to enhance economic benefits by increasing animal body weight through muscle and fat accumulation (12). Pech Cervantes and colleagues discovered that dietary grape pomace improves sheep meat tenderness and reduces cooking loss (13). In the current experiment, the average daily gain of Dorper × Hu Sheep fed with complete GP-based pelleted feed moderately increased. The differences between our results and previous study may be attributed to factors including the level of GP addition, diet composition, sheep breed, and feeding environment. Currently, no studies have suggested that GP adversely affects animal production performance (14).

Blood biochemical parameters are indicators of nutrient metabolism to monitor the health status of animals and understand nutrient utilization (15). Total protein, albumin, globulin and urea nitrogen can reflect the utilization of protein in the body (16). Globulin, synthesized in the liver, is vital for the body’s immune defense, increasing resistance to viruses and other microorganisms (17). Urea nitrogen, a byproduct of protein metabolism, suggests enhanced protein catabolism, reduced nitrogen deposition, or abnormal kidney function when elevated, indicating potential health issues (18–20). The supplementation of 1.5% purified condensed tannins had no adverse effects on the body of the sheep (21). In this study, increases in urea nitrogen, total protein, albumin, globulin, and total cholesterol were observed with higher GP levels, suggesting improved protein digestion and absorption and enhanced immune responses in Dorper × Hu sheep.

Antioxidant capacity plays a vital role in health maintenance. Antioxidant enzyme can significantly reduce tissue oxidative stress and resists lipid peroxidation, marking it as a key indicator of tissue antioxidant capacity. Malondialdehyde, a byproduct of lipid peroxidation, serves as a critical marker in this process. The inclusion of 4–8% grape residue did not significantly influence the antioxidant indices of laying hens, including T-SOD, T-AOC, GSH-Px, and others (22). The inclusion of 5 and 10% grape dregs in the diets of broilers enhanced the antioxidant status of the poultry, leading to increased alpha-tocopherol levels and reduced plasma iron concentrations. Notably, plasma glutathione levels remained unaffected (23). This study found a decrease in malondialdehyde levels with higher GP levels, aligning with findings by Bocsan and co-workers (24). In this study, the activity of glutathione peroxidase exhibited a significant increasing trend with the addition of grape residue. Plasma glutathione serves as a natural intracellular scavenger of reactive oxygen species, playing a crucial role in cellular defense mechanisms and protecting tissues from oxidative damage (25). These trends suggest that bioactive substances in GP can scavenge free radicals and inhibit lipid peroxidation through the body’s antioxidant enzyme activity (26). Thus, incorporating GP into the diet enhances internal oxidative stress management, eradicates free radicals, and sustains redox capacity, improving the antioxidant defense of the mutton sheep immune system.

The variety and concentration of amino acids in muscle decisively influence the meat’s nutritional value and are crucial in evaluating protein quality (27). The concentrations of Thr, Val, Met, Cys, and His in the thigh muscle of Ross308 broilers were significantly elevated when the diet was supplemented with 1 and 3% grape dregs, which aligns with the findings of this study (28). This increase may result from the utilization and deposition of amino acids abundant in GP during the protein metabolism of Dorper × Hu sheep. Umami is recognized as the fifth basic taste that humans can perceive during the course of eating. Glutamic acid and glycine are key umami amino acids in meat, with glutamic acid being the primary contributor to umami flavor. This taste is further enhanced by the synergistic interaction between free amino acids and nucleotides (29). In this study, glutamic acid content was highest in the 15% GP treatment, likely due to the efficient deposition effect of lower GP levels. The addition of 20% GP appeared to enhance the unique meat flavor in Dorper × Hu sheep.

Rumen microorganisms play a significant role in animal nutrition, health, and immune function (30). This experiment demonstrated that supplementing the diet with 15% or 20% grape pomace (GP) did not significantly affect the overall diversity and richness of the rumen microbiota in Dorper-Hu sheep. However, GP supplementation led to a declining trend in the Chao1 index, suggesting a potential reduction in ruminal bacterial diversity, which aligns with previous findings (31–33). These results indicate that dietary composition is a key factor influencing the diversity of the rumen flora in ruminants.

*Bacteroidetes and Firmicutes* are dominant in the rumen of ruminants (34, 35). In this experiment, Bacteroidetes, Firmicutes, Proteobacteria, Verrucomicrobia, and Spirochaetes were identified as the most abundant groups. The Bacteroidetes and Firmicutes are the primary dominant groups. A mutualistic symbiotic relationship exists between Bacteroidetes and Firmicutes. A mutualistic symbiotic relationship exists between them; Bacteroidetes primarily degrade carbohydrates, proteins, and polysaccharides, while Firmicutes, rich in fiber-degrading bacteria, promote the digestion and absorption of fibrous materials in ruminants (36). In this experiment, higher GP levels were associated with an increased relative abundance of Firmicutes, suggesting that GP positively stimulates their proliferation in the rumen. Supplementation with or 20% GP significantly reduced the relative abundance of Spirochaetes, indicating a beneficial role of GP in suppressing harmful flora. Furthermore, an increase was observed in the number of Proteobacteria, which plays a role in the nitrogen cycle and the metabolism and synthesis of nutrients and vitamins. This suggests that GP promotes the growth and reproduction of Proteobacteria, potentially because the phenolic acids in GP influence the rumen microbiota.

*Prevotella* is involved in both protein degradation and, through collaboration with fibrolytic bacteria, the breakdown of dietary fiber. Our results showed that adding 15% or 20% GP reduced the relative abundance of *Prevotella*, likely due to the inhibitory effect of GP tannins on this genus and on Treponema (37). This implies that GP can reduce potentially harmful bacteria and help maintain rumen microbial balance, thus lowering disease risk. Additionally, the relative abundance of Succiniclasticum (a fibrolytic bacterium also involved in protein degradation) increased with GP levels, a trend that paralleled the increase in blood urea nitrogen in the sheep.

## 5 Conclusion

In summary, dietary supplementation with 15 and 20% GP improved the average daily gain, serum levels of total protein and albumin, and the total antioxidant capacity in Dorper × Hu sheep. Furthermore, GP significantly increased the content of essential amino acids in the longissimus dorsi muscle and modified the rumen bacterial community composition. This study demonstrates the feasibility of utilizing GP as a roughage source in complete pelleted feed. For optimal production outcomes, a 15% GP inclusion level is recommended, as it yielded the best feed conversion ratio along with the highest levels of glutamic acid and catalase activity.

## Data Availability

The data that support the findings of this study are available from the corresponding author upon reasonable request. The raw data on microbial diversity of Dorper × Hu sheep rumen have been submitted to NCBI, with accession numbers PRJNA1197673.
